# P07 Impact of menopausal status and recurrent urinary tract infections (UTIs) on symptoms, severity, and daily life: findings from an online survey of women reporting a recent UTI

**DOI:** 10.1093/jacamr/dlad077.011

**Published:** 2023-08-02

**Authors:** Leigh Sanyaolu, Emily Cooper, Brieze Read, Haroon Ahmed, Donna M Lecky

**Affiliations:** Division of Population Medicine, Cardiff University, Cardiff, UK; Primary Care and Interventions Unit, UKHSA, Twyver House, Gloucester, GL1 1DQ, UK; Primary Care and Interventions Unit, UKHSA, Twyver House, Gloucester, GL1 1DQ, UK; Division of Population Medicine, Cardiff University, Cardiff, UK; Primary Care and Interventions Unit, UKHSA, Twyver House, Gloucester, GL1 1DQ, UK; School of Medicine, Cardiff University, Cardiff, UK

## Abstract

**Background:**

Current UKHSA urinary tract infection (UTI) guidance advises empirical antibiotics if two of the following symptoms are present: cloudy urine, dysuria, and new onset nocturia. Hormonal changes during menopause may affect UTI symptoms. Qualitative studies suggest women with recurrent UTIs may present with different UTI symptoms. This study aims to assess whether menopausal status and a history of recurrent UTIs affects UTI symptoms in women.

**Methods:**

An e-survey was conducted between 13 March 2021 and 13 April 2021. Women aged 16 years or older with a history of a UTI in the past year were eligible for inclusion. We defined menopause as those aged 45–64 years; pre-menopause as those less than 45 years; and post-menopause as those 65 years and older. Recurrent UTIs were defined as three or more UTIs in the last year. Data were weighted to be representative of the UK population. Crude and adjusted ORs were estimated using logistic regression.

**Results:**

A total of 1096 women reported a UTI in the last year. There were significant differences in UTI symptoms based on menopausal status and the presence of recurrent UTIs. Post-menopausal women were more likely to self-report new or worsening incontinence (OR 2.76, 95% CI 1.50, 5.09) with an acute UTI compared with pre-menopausal women. Menopausal women were also more likely to self-report new or worsening incontinence (OR 1.66, 95% CI 1.06, 2.62) and nocturia (OR 1.43, 95% CI 1.05, 1.96) with an acute UTI compared with pre-menopausal women. Women with recurrent UTIs were less likely to self-report dysuria with an acute UTI but reported more severe symptoms (OR 1.93 95% CI 1.37, 2.73) and a greater impact on daily life (OR 1.68, 95% CI 1.19, 2.37) compared with women without recurrent UTIs.

**Conclusions:**

This survey provides evidence that acute UTIs present differently based on menopausal status and in women with recurrent UTIs. This has implications for the diagnosis and treatment of acute UTIs in these patient populations and suggests that a more tailored approach may be required that takes account of menopausal status and history of recurrent UTIs. It is important healthcare professionals are aware of these differences when assessing women presenting with an acute UTI.

